# Bis(4-acet­oxy-*N*,*N*-di­methyl­tryptammonium) fumarate: a new crystalline form of psilacetin, an alternative to psilocybin as a psilocin prodrug

**DOI:** 10.1107/S2056989019007370

**Published:** 2019-05-31

**Authors:** Andrew R. Chadeayne, James A. Golen, David R. Manke

**Affiliations:** a CaamTech, LLC, 58 East Sunset Way, Suite 209, Issaquah, WA 98027, USA; b Department of Chemistry and Biochemistry, University of Massachusetts Dartmouth, 285 Old Westport Road, North Dartmouth, MA 02747, USA

**Keywords:** crystal structure, tryptamines, hydrogen bonding

## Abstract

The title compound (systematic name: bis­{2-[4-(acet­yloxy)-1*H*-indol-3-yl]ethan-1-aminium} but-2-enedioate), 2C_14_H_19_N_2_O_2_^+^·C_4_H_2_O_4_^2−^, has a single protonated psilacetin cation and one half of a fumarate dianion in the asymmetric unit. There are N—H⋯O hydrogen bonds between the ammonium H atoms and the fumarate O atoms, as well as N—H⋯O hydrogen bonds between the indole H atoms and the fumarate O atoms. The hydrogen bonds hold the ions together in infinite one-dimensional chains along [111].

## Abstract 2

The title compound has a single protonated psilacetin cation and one half of a fumarate dianion in the asymmetric unit. The ions are held together through N—H⋯O hydrogen bonds in infinite one-dimensional chains along [111].

## Chemical context   

Psychedelic agents have received a great deal of inter­est lately as potential pharmaceuticals to treat mood disorders, including depression and post traumatic stress disorder (PTSD) (Carhart-Harris & Goodwin, 2017 ▸). Psilocybin, a naturally occurring tryptamine derivative found in ‘magic’ mushrooms, is a prodrug of psilocin. When consumed orally, psilocybin hydrolyzes to generate psilocin, a serotonin-2a agonist, producing mood-altering or ‘psychedelic’ effects (Dinis-Oliveira, 2017 ▸). Like psilocybin, psilacetin serves as a prodrug of psilocin. Compared to psilocybin, psilacetin is easier and less expensive to synthesize. This suggests that administering psilacetin (instead of psilocybin) represents a better means of delivery for the active psilocin. Psilacetin was first reported in 1999 by Nichols and co-workers (Nichols & Frescas, 1999 ▸), generally producing the mol­ecule as its crystalline fumarate salt. Psilacetin was structurally characterized earlier this year (Chadeayne *et al.*, 2019 ▸). Herein we report the structure of a new crystalline form of psilacetin, in which two psilacetin mol­ecules are protonated, and charge-balanced by one fumarate dianion.
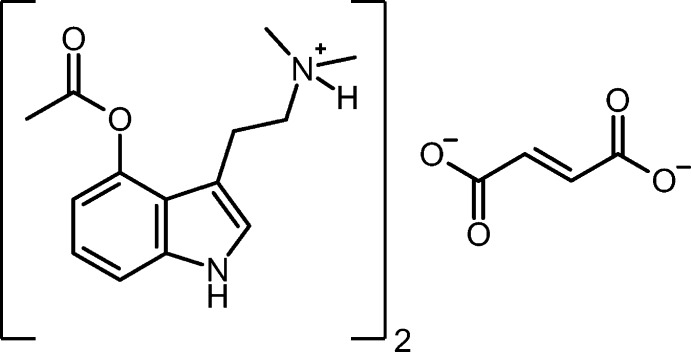


## Structural commentary   

**Figure 1:**
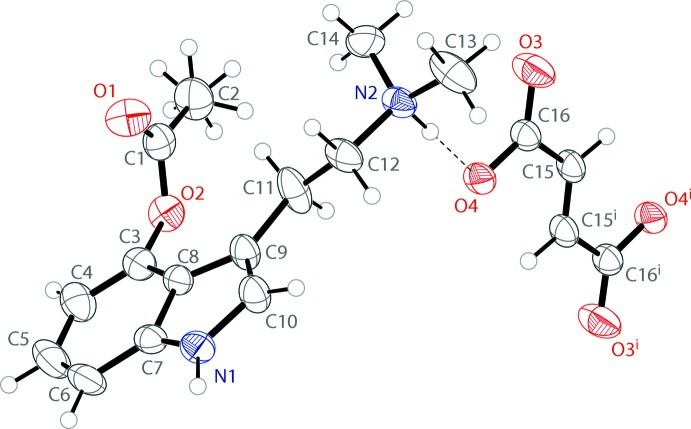
The mol­ecular structure of bis­(4-acet­oxy-*N*,*N*-di­methyl­tryptammonium) fumarate, showing the atomic labeling. Displacement ellipsoids are drawn at the 50% probability level. Hydrogen bonds are shown as dashed lines. Symmetry code: (i) 2 − *x*, 1 − *y*, 2 − *z*.

The mol­ecular structure of bis­(4-acet­oxy-*N*,*N*-di­methyl­tryptammonium) fumarate is shown in Fig. 1 ▸. The cation possesses a near-planar indole, with a mean deviation from planarity of 0.04 Å. The acetate on the 4-position of the indole is approximately perpendicular, with the angles between the indole and acetate planes being 100.85 (1)°. Half of a fumarate ion is present in the asymmetric unit, with the full dianion produced through inversion. The fumarate shows a near planar *trans* configuration with a deviation from planarity of 0.019 Å. A series of N—H⋯O hydrogen bonds hold the ions together in the solid state.

## Supra­molecular features   

**Figure 2:**
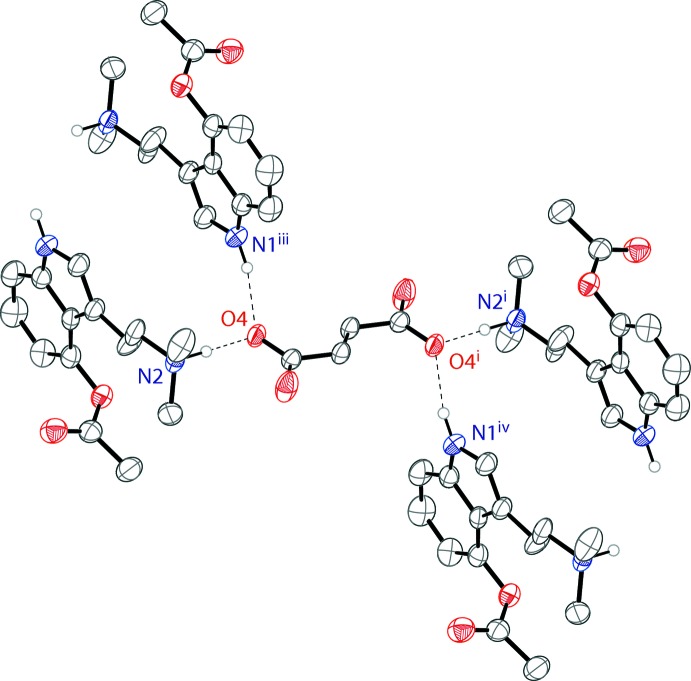
The hydrogen bonding of the fumarate ion in the structure of the title compound. Displacement ellipsoids are drawn at the 50% probability level. Hydrogen atoms not involved in hydrogen bonds are omitted for clarity. Symmetry codes: (i) 2 − *x*, 1 − *y*, 2 − *z*, (iii) 1 − *x*, 1 + *y*, 1 + *z*, (iv) 1 − *x*, −*y*, 1 − *z*.

**Figure 3:**
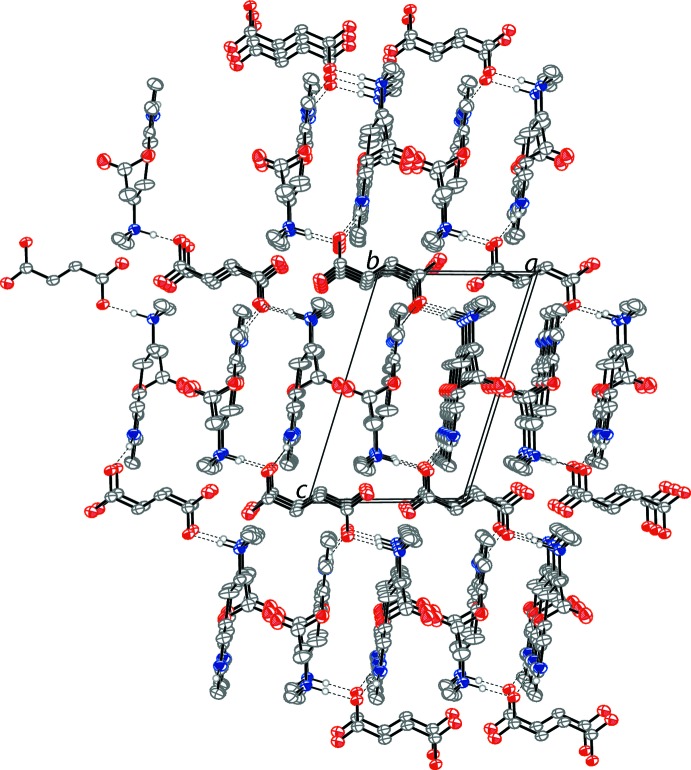
The crystal packing of the title compound, viewed along the *b* axis. The N—H⋯O bonds (Table 1 ▸) are shown as dashed lines. Displacement ellipsoids are drawn at the 50% probability level. Hydrogen atoms not involved in hydrogen bonding are omitted for clarity

The 4-acet­oxy-*N*,*N*-di­methyl­tryptammonium cations and fumarate dianions are held together in an infinite one-dimensional chain through N—H⋯O hydrogen bonds (Table 1 ▸) along the [111] direction. The anionic oxygen of the carb­oxy­lic acid possesses a hydrogen bond with the ammonium proton of the psilacetin mol­ecule. Each of these oxygens also forms a hydrogen bond with the hydrogen of an indole nitro­gen of a different psilacetin cation. Both anionic oxygens of the fumarate dianions form the same hydrogen-bonding inter­actions, generated through symmetry. The hydrogen-bonding inter­actions of a single fumarate dianion are shown in Fig. 2 ▸. The packing of the compound is shown in Fig. 3 ▸.

## Database survey   

We recently reported a closely related structure in which one 4-acet­oxy-*N*,*N*-di­methyl­tryptammonium cation is charge balanced by one 3-carb­oxy­acrylate anion (Chadeayne *et al.*, 2019 ▸). The structure reported here has the same 4-acet­oxy-*N*,*N*-di­methyl­tryptammonium cation, two of which are charge-balanced by a single fumarate dianion. The bond distances and angles observed in the compound reported here are consistent with our prior report. The two other reported 4-substituted tryptamine structures are those of the naturally occurring products of ‘magic’ mushrooms – psilocybin, C_12_H_16_N_2_PO_4_ (Weber & Petcher, 1974 ▸) and psilocin, C_12_H_16_N_2_O (Petcher & Weber, 1974 ▸). Psilocybin is the 4-phosphate-substituted variation of *N*,*N*-di­methyl­tryptamine, and exists as an ammonium/phosphate zwitterion in the solid state. Psilocin, 4-hy­droxy-*N*,*N*-di­methyl­tryptamine, is believed to be a statistical mixture of a neutral mol­ecule and an ammonium/phenoxide zwitterion. In both cases, the tryptamine components are structurally very similar to the title compound, but their arrangements in the solid state are substanti­ally different as there are no counter-ions present.

## Synthesis and crystallization   

A commercial sample (The Indole Shop) of 4-acet­oxy-*N*,*N*-di­methyl­tryptamine fumarate (100 mg, 0.16 mmol) was dissolved in 10 mL of water and treated with one equivalent of lead(II) acetate­(53 mg, 0.16 mmol). Lead(II) fumarate precipitated and was filtered [the presence of lead(II) fumarate was confirmed by the unit cell of the precipitate]. Water was removed *in vacuo* and the resulting residue was picked up in acetone and filtered. The filtrate was allowed to evaporate slowly, resulting in single crystals suitable for X-ray analysis.

## Refinement   

Crystal data, data collection and structure refinement details are summarized in Table 2 ▸. The methyl hydrogens on C2 were disordered over two positions and were refined at 50% occupancy with the C–C–H planes set at 60^o^ to each other. The H atoms on N1 and N2 were found in the difference-Fourier map and refined freely. H atoms were placed in calculated positions (C—H = 0.95–0.99 Å) and refined as riding with *U*_iso_(H) = 1.5*U*_eq_(C-meth­yl) and 1.2*U*_eq_(C) for all other H atoms.

## Supplementary Material

Crystal structure: contains datablock(s) I. DOI: 10.1107/S2056989019007370/ff2159sup1.cif

Structure factors: contains datablock(s) I. DOI: 10.1107/S2056989019007370/ff2159Isup2.hkl

Supporting information file. DOI: 10.1107/S2056989019007370/ff2159Isup3.cml

CCDC reference: 1917404

Additional supporting information:  crystallographic information; 3D view; checkCIF report

## Figures and Tables

**Table 1:** Hydrogen-bond geometry (Å, °)

*D*—H⋯*A*	*D*—H	H⋯*A*	*D*⋯*A*	*D*—H⋯*A*
N1—H1⋯O4^ii^	0.90 (2)	1.91 (2)	2.786 (2)	165 (2)
N2—H2⋯O4	0.99 (2)	1.61 (2)	2.607 (2)	179 (2)

Symmetry code: (ii) 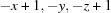.

**Table 2:** Experimental details

Crystal data	
Chemical formula	2C_14_H_19_N_2_O_2_^+^·C_4_H_2_O_4_^2−^
*M*_r_	608.68
Crystal system, space group	Triclinic, *P* 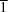
Temperature (K)	200
*a*, *b*, *c* (Å)	8.3965 (13), 8.9879 (14), 12.0126 (16)
α, β, γ (°)	101.730 (5), 100.818 (5), 112.463 (5)
*V* (Å^3^)	784.2 (2)
*Z*	1
Radiation type	Mo *K*α
μ (mm^−1^)	0.09
Crystal size (mm)	0.19 × 0.16 × 0.13

Data collection	
Diffractometer	Bruker D8 Venture CMOS
Absorption correction	Multi-scan (*SADABS*; Bruker, 2016 ▸)
*T*_min_, *T*_max_	0.714, 0.745
No. of measured, independent and observed [*I* > 2σ(*I*)] reflections	21581, 2877, 2087
*R*_int_	0.056
(sin θ/λ)_max_ (Å^−1^)	0.604

Refinement	
*R*[*F* ^2^ > 2σ(*F* ^2^)], *wR*(*F* ^2^), *S*	0.045, 0.110, 1.03
No. of reflections	2877
No. of parameters	210
H-atom treatment	H atoms treated by a mixture of independent and constrained refinement
Δρ_max_, Δρ_min_ (e Å^−3^)	0.26, −0.20

Computer programs: *APEX3* and *SAINT* (Bruker, 2016 ▸), *SHELXT2014* (Sheldrick, 2015 ▸), *SHELXL97* (Sheldrick, 2008 ▸) and *OLEX2* (Dolomanov *et al.*, 2009 ▸).

## supplementary crystallographic information

### Crystal data

**Table:**

2C_14_H_19_N_2_O_2_^+^·C_4_H_2_O_4_^2^^−^	*Z* = 1
*M**_r_* = 608.68	*F*(000) = 324
Triclinic, *P*1	*D*_x_ = 1.289 Mg m^−^^3^
*a* = 8.3965 (13) Å	Mo *K*α radiation, λ = 0.71073 Å
*b* = 8.9879 (14) Å	Cell parameters from 6407 reflections
*c* = 12.0126 (16) Å	θ = 3.3–25.1°
α = 101.730 (5)°	µ = 0.09 mm^−^^1^
β = 100.818 (5)°	*T* = 200 K
γ = 112.463 (5)°	BLOCK, colourless
*V* = 784.2 (2) Å^3^	0.19 × 0.16 × 0.13 mm

### Data collection

**Table:**

Bruker D8 Venture CMOS diffractometer	2087 reflections with *I* > 2σ(*I*)
φ and ω scans	*R*_int_ = 0.056
Absorption correction: multi-scan (SADABS; Bruker, 2016)	θ_max_ = 25.4°, θ_min_ = 3.3°
*T*_min_ = 0.714, *T*_max_ = 0.745	*h* = −10→10
21581 measured reflections	*k* = −10→10
2877 independent reflections	*l* = −14→14

### Refinement

**Table:**

Refinement on *F*^2^	Hydrogen site location: mixed
Least-squares matrix: full	H atoms treated by a mixture of independent and constrained refinement
*R*[*F*^2^ > 2σ(*F*^2^)] = 0.045	*w* = 1/[σ^2^(*F*_o_^2^) + (0.0387*P*)^2^ + 0.3852*P*] where *P* = (*F*_o_^2^ + 2*F*_c_^2^)/3
*wR*(*F*^2^) = 0.110	(Δ/σ)_max_ < 0.001
*S* = 1.03	Δρ_max_ = 0.26 e Å^−^^3^
2877 reflections	Δρ_min_ = −0.20 e Å^−^^3^
210 parameters	Extinction correction: SHELXL97 (Sheldrick, 2008), Fc^*^=kFc[1+0.001xFc^2^λ^3^/sin(2θ)]^-1/4^
0 restraints	Extinction coefficient: 0.050 (4)

### Special details

**Table:**

Geometry. All esds (except the esd in the dihedral angle between two l.s. planes) are estimated using the full covariance matrix. The cell esds are taken into account individually in the estimation of esds in distances, angles and torsion angles; correlations between esds in cell parameters are only used when they are defined by crystal symmetry. An approximate (isotropic) treatment of cell esds is used for estimating esds involving l.s. planes.

### Fractional atomic coordinates and isotropic or equivalent isotropic displacement parameters (Å^2^)

**Table:**

	*x*	*y*	*z*	*U*_iso_*/*U*_eq_	Occ. (<1)
O1	0.0322 (2)	0.33780 (19)	0.50722 (14)	0.0539 (4)	
O2	0.30616 (18)	0.46535 (16)	0.48485 (12)	0.0413 (4)	
O3	0.6497 (2)	0.3361 (2)	1.04226 (12)	0.0597 (5)	
O4	0.69101 (17)	0.29711 (17)	0.86294 (11)	0.0386 (4)	
N1	0.2368 (2)	−0.0732 (2)	0.29315 (15)	0.0412 (4)	
N2	0.3416 (2)	0.1243 (2)	0.79890 (13)	0.0375 (4)	
C1	0.2567 (4)	0.5920 (3)	0.6569 (2)	0.0578 (6)	
H1A	0.3818	0.6670	0.6650	0.087*	0.5
H1B	0.1847	0.6558	0.6561	0.087*	0.5
H1C	0.2531	0.5457	0.7240	0.087*	0.5
H1D	0.1646	0.5787	0.6984	0.087*	0.5
H1E	0.3617	0.5899	0.7073	0.087*	0.5
H1F	0.2933	0.7000	0.6394	0.087*	0.5
C2	0.1817 (3)	0.4512 (3)	0.54365 (18)	0.0406 (5)	
C3	0.2616 (3)	0.3380 (2)	0.37872 (17)	0.0367 (5)	
C4	0.2330 (3)	0.3748 (3)	0.27365 (19)	0.0482 (6)	
H4	0.2329	0.4804	0.2735	0.058*	
C5	0.2040 (3)	0.2580 (3)	0.1667 (2)	0.0572 (7)	
H5	0.1839	0.2852	0.0945	0.069*	
C6	0.2041 (3)	0.1050 (3)	0.16395 (18)	0.0491 (6)	
H6	0.1855	0.0261	0.0912	0.059*	
C7	0.2325 (2)	0.0689 (3)	0.27162 (16)	0.0361 (5)	
C8	0.2611 (2)	0.1833 (2)	0.38128 (15)	0.0324 (4)	
C9	0.2860 (3)	0.1039 (2)	0.47124 (16)	0.0378 (5)	
C10	0.2697 (3)	−0.0505 (3)	0.41319 (17)	0.0428 (5)	
H10	0.2796	−0.1313	0.4506	0.051*	
C11	0.3232 (4)	0.1765 (3)	0.60302 (17)	0.0552 (7)	
H11A	0.4523	0.2569	0.6377	0.066*	
H11B	0.2518	0.2408	0.6160	0.066*	
C12	0.2802 (3)	0.0483 (3)	0.66691 (16)	0.0446 (6)	
H12A	0.3380	−0.0264	0.6453	0.054*	
H12B	0.1482	−0.0222	0.6407	0.054*	
C13	0.3087 (4)	−0.0104 (3)	0.8580 (2)	0.0623 (7)	
H13A	0.3654	−0.0819	0.8298	0.094*	
H13B	0.1786	−0.0794	0.8389	0.094*	
H13C	0.3603	0.0415	0.9443	0.094*	
C14	0.2660 (3)	0.2400 (4)	0.84318 (19)	0.0610 (7)	
H14A	0.3073	0.2793	0.9303	0.092*	
H14B	0.1341	0.1804	0.8159	0.092*	
H14C	0.3063	0.3371	0.8128	0.092*	
C15	0.9426 (3)	0.4767 (2)	1.03029 (16)	0.0346 (5)	
H15	0.9873	0.5193	1.1146	0.042*	
C16	0.7463 (3)	0.3612 (2)	0.97637 (16)	0.0340 (5)	
H1	0.240 (3)	−0.159 (3)	0.242 (2)	0.055 (7)*	
H2	0.475 (3)	0.191 (3)	0.8236 (18)	0.048 (6)*	

### Atomic displacement parameters (Å^2^)

**Table:**

	*U*^11^	*U*^22^	*U*^33^	*U*^12^	*U*^13^	*U*^23^
O1	0.0506 (10)	0.0427 (9)	0.0626 (10)	0.0148 (8)	0.0223 (8)	0.0098 (8)
O2	0.0381 (8)	0.0330 (8)	0.0418 (8)	0.0099 (6)	0.0069 (6)	0.0048 (6)
O3	0.0445 (9)	0.0788 (12)	0.0310 (8)	0.0068 (8)	0.0124 (7)	0.0053 (8)
O4	0.0332 (8)	0.0447 (8)	0.0248 (7)	0.0120 (6)	0.0032 (6)	−0.0008 (6)
N1	0.0449 (11)	0.0422 (11)	0.0307 (9)	0.0198 (9)	0.0081 (8)	0.0003 (8)
N2	0.0323 (9)	0.0398 (10)	0.0267 (8)	0.0065 (8)	0.0070 (7)	0.0022 (7)
C1	0.0716 (17)	0.0518 (14)	0.0449 (13)	0.0323 (13)	0.0071 (12)	0.0028 (11)
C2	0.0472 (13)	0.0356 (12)	0.0406 (12)	0.0208 (11)	0.0097 (10)	0.0125 (9)
C3	0.0318 (11)	0.0356 (11)	0.0347 (11)	0.0086 (9)	0.0082 (8)	0.0083 (9)
C4	0.0514 (14)	0.0464 (13)	0.0463 (13)	0.0165 (11)	0.0143 (10)	0.0230 (11)
C5	0.0671 (16)	0.0699 (17)	0.0386 (13)	0.0257 (13)	0.0204 (11)	0.0283 (12)
C6	0.0501 (13)	0.0634 (16)	0.0291 (11)	0.0189 (12)	0.0169 (10)	0.0114 (10)
C7	0.0287 (10)	0.0423 (12)	0.0308 (10)	0.0106 (9)	0.0097 (8)	0.0070 (9)
C8	0.0254 (10)	0.0364 (11)	0.0270 (9)	0.0082 (8)	0.0050 (7)	0.0055 (8)
C9	0.0436 (12)	0.0359 (11)	0.0269 (10)	0.0170 (9)	0.0019 (8)	0.0036 (8)
C10	0.0511 (13)	0.0416 (12)	0.0306 (11)	0.0217 (10)	0.0034 (9)	0.0055 (9)
C11	0.0909 (19)	0.0408 (13)	0.0269 (11)	0.0334 (13)	−0.0004 (11)	0.0031 (9)
C12	0.0386 (12)	0.0459 (13)	0.0265 (10)	0.0036 (10)	0.0053 (9)	−0.0018 (9)
C13	0.0717 (17)	0.0533 (15)	0.0392 (12)	0.0026 (13)	0.0183 (12)	0.0149 (11)
C14	0.0548 (15)	0.091 (2)	0.0379 (12)	0.0421 (14)	0.0129 (11)	0.0014 (12)
C15	0.0373 (11)	0.0371 (11)	0.0228 (9)	0.0151 (9)	0.0025 (7)	0.0037 (8)
C16	0.0372 (11)	0.0350 (11)	0.0267 (10)	0.0159 (9)	0.0065 (8)	0.0056 (8)

### Geometric parameters (Å, º)

**Table:**

O1—C2	1.200 (2)	C5—H5	0.9500
O2—C2	1.349 (2)	C5—C6	1.369 (3)
O2—C3	1.405 (2)	C6—H6	0.9500
O3—C16	1.228 (2)	C6—C7	1.396 (3)
O4—C16	1.282 (2)	C7—C8	1.412 (3)
N1—C7	1.365 (3)	C8—C9	1.437 (3)
N1—C10	1.373 (3)	C9—C10	1.362 (3)
N1—H1	0.90 (2)	C9—C11	1.506 (3)
N2—C12	1.493 (2)	C10—H10	0.9500
N2—C13	1.488 (3)	C11—H11A	0.9900
N2—C14	1.475 (3)	C11—H11B	0.9900
N2—H2	0.99 (2)	C11—C12	1.480 (3)
C1—H1A	0.9800	C12—H12A	0.9900
C1—H1B	0.9800	C12—H12B	0.9900
C1—H1C	0.9800	C13—H13A	0.9800
C1—H1D	0.9800	C13—H13B	0.9800
C1—H1E	0.9800	C13—H13C	0.9800
C1—H1F	0.9800	C14—H14A	0.9800
C1—C2	1.489 (3)	C14—H14B	0.9800
C3—C4	1.370 (3)	C14—H14C	0.9800
C3—C8	1.396 (3)	C15—C15^i^	1.309 (4)
C4—H4	0.9500	C15—H15	0.9500
C4—C5	1.397 (3)	C15—C16	1.494 (3)
			
C2—O2—C3	118.62 (15)	C5—C6—C7	117.8 (2)
C7—N1—C10	108.50 (17)	C7—C6—H6	121.1
C7—N1—H1	126.7 (15)	N1—C7—C6	129.45 (19)
C10—N1—H1	123.8 (15)	N1—C7—C8	107.93 (17)
C12—N2—H2	107.7 (12)	C6—C7—C8	122.6 (2)
C13—N2—C12	110.37 (16)	C3—C8—C7	117.04 (17)
C13—N2—H2	105.4 (12)	C3—C8—C9	136.10 (17)
C14—N2—C12	114.55 (17)	C7—C8—C9	106.85 (17)
C14—N2—C13	111.26 (18)	C8—C9—C11	127.06 (18)
C14—N2—H2	107.1 (12)	C10—C9—C8	106.02 (16)
H1A—C1—H1B	109.5	C10—C9—C11	126.91 (18)
H1A—C1—H1C	109.5	N1—C10—H10	124.7
H1A—C1—H1D	141.1	C9—C10—N1	110.70 (19)
H1A—C1—H1E	56.3	C9—C10—H10	124.7
H1A—C1—H1F	56.3	C9—C11—H11A	108.7
H1B—C1—H1C	109.5	C9—C11—H11B	108.7
H1B—C1—H1D	56.3	H11A—C11—H11B	107.6
H1B—C1—H1E	141.1	C12—C11—C9	114.06 (17)
H1B—C1—H1F	56.3	C12—C11—H11A	108.7
H1C—C1—H1D	56.3	C12—C11—H11B	108.7
H1C—C1—H1E	56.3	N2—C12—H12A	109.0
H1C—C1—H1F	141.1	N2—C12—H12B	109.0
H1D—C1—H1E	109.5	C11—C12—N2	112.99 (16)
H1D—C1—H1F	109.5	C11—C12—H12A	109.0
H1E—C1—H1F	109.5	C11—C12—H12B	109.0
C2—C1—H1A	109.5	H12A—C12—H12B	107.8
C2—C1—H1B	109.5	N2—C13—H13A	109.5
C2—C1—H1C	109.5	N2—C13—H13B	109.5
C2—C1—H1D	109.5	N2—C13—H13C	109.5
C2—C1—H1E	109.5	H13A—C13—H13B	109.5
C2—C1—H1F	109.5	H13A—C13—H13C	109.5
O1—C2—O2	122.94 (19)	H13B—C13—H13C	109.5
O1—C2—C1	126.3 (2)	N2—C14—H14A	109.5
O2—C2—C1	110.81 (19)	N2—C14—H14B	109.5
C4—C3—O2	118.17 (19)	N2—C14—H14C	109.5
C4—C3—C8	120.95 (19)	H14A—C14—H14B	109.5
C8—C3—O2	120.68 (17)	H14A—C14—H14C	109.5
C3—C4—H4	119.8	H14B—C14—H14C	109.5
C3—C4—C5	120.4 (2)	C15^i^—C15—H15	117.7
C5—C4—H4	119.8	C15^i^—C15—C16	124.7 (2)
C4—C5—H5	119.4	C16—C15—H15	117.7
C6—C5—C4	121.2 (2)	O3—C16—O4	124.80 (18)
C6—C5—H5	119.4	O3—C16—C15	118.60 (16)
C5—C6—H6	121.1	O4—C16—C15	116.59 (16)
			
O2—C3—C4—C5	174.36 (19)	C6—C7—C8—C3	−0.6 (3)
O2—C3—C8—C7	−173.84 (16)	C6—C7—C8—C9	−179.64 (19)
O2—C3—C8—C9	4.9 (3)	C7—N1—C10—C9	0.4 (2)
N1—C7—C8—C3	−179.97 (16)	C7—C8—C9—C10	−0.7 (2)
N1—C7—C8—C9	1.0 (2)	C7—C8—C9—C11	179.2 (2)
C2—O2—C3—C4	107.9 (2)	C8—C3—C4—C5	−0.5 (3)
C2—O2—C3—C8	−77.2 (2)	C8—C9—C10—N1	0.2 (2)
C3—O2—C2—O1	−2.8 (3)	C8—C9—C11—C12	159.8 (2)
C3—O2—C2—C1	176.73 (17)	C9—C11—C12—N2	172.04 (19)
C3—C4—C5—C6	−0.3 (4)	C10—N1—C7—C6	179.8 (2)
C3—C8—C9—C10	−179.5 (2)	C10—N1—C7—C8	−0.8 (2)
C3—C8—C9—C11	0.3 (4)	C10—C9—C11—C12	−20.4 (4)
C4—C3—C8—C7	0.9 (3)	C11—C9—C10—N1	−179.7 (2)
C4—C3—C8—C9	179.6 (2)	C13—N2—C12—C11	−175.2 (2)
C4—C5—C6—C7	0.6 (3)	C14—N2—C12—C11	58.3 (3)
C5—C6—C7—N1	179.1 (2)	C15^i^—C15—C16—O3	−174.9 (3)
C5—C6—C7—C8	−0.2 (3)	C15^i^—C15—C16—O4	4.1 (4)

Symmetry code: (i) −*x*+2, −*y*+1, −*z*+2.

### Hydrogen-bond geometry (Å, º)

**Table:**

*D*—H···*A*	*D*—H	H···*A*	*D*···*A*	*D*—H···*A*
N1—H1···O4^ii^	0.90 (2)	1.91 (2)	2.786 (2)	165 (2)
N2—H2···O4	0.99 (2)	1.61 (2)	2.607 (2)	179 (2)

Symmetry code: (ii) −*x*+1, −*y*, −*z*+1.
